# Homologous recombination mRNAs (*RAD21*, *RAD50* and *BARD1*) have a potentially poor prognostic role in *ERBB2*-low bladder cancer patients

**DOI:** 10.1038/s41598-023-38923-y

**Published:** 2023-07-20

**Authors:** Nada Albarakati, Hanin Al-Ghamdi, Batla Al-Sowayan, Alaa Alshareeda

**Affiliations:** 1 Department of Blood and Cancer Research, King Abdullah International Medical Research Center, Jeddah, Kingdom of Saudi Arabia; 2 Department of Blood and Cancer Research, King Abdullah International Medical Research Center, Riyadh, Saudi Arabia; 3 King Saud Bin Abdulaziz University for Health Sciences, Ministry of the National Guard-Health Affairs, Riyadh, Saudi Arabia

**Keywords:** Cancer, Urological cancer, Bladder cancer

## Abstract

Human epidermal growth factor receptor 2 (HER2/*ERBB2*) factor is known to be implicated in many malignancies and the potential of it as a prognostic biomarker was reported years ago. Molecular subtypes of HER2/*ERBB2* negative and positive with distinct clinical outcomes have been identified in recent years; however, it is still under investigation for bladder cancer. This study evaluates the biological and prognostic significance of *RAD21*, *RAD50* and *BARD1* (homologous recombination biomarkers) mRNA levels with *ERBB2* low and high expression to explore their impact on bladder cancer patient survival and cancer aggressiveness. The expression of *ERBB2*, *RAD21*, *RAD50* and *BARD1* mRNA levels was assessed in The Cancer Genome Atlas (TCGA) bladder cancer dataset along with four validation cohorts. Outcome analysis was evaluated using disease-free survival (DFS) and overall survival (OS). Univariate and multivariate analysis were used to evaluate the relationship between *RAD21*, *RAD50*, *BARD1* and *ERBB2* expression and clinicopathological variables. A significant increase in mRNA expression levels of *RAD21*, *RAD50* and *BARD1* was noticed in *ERBB2*-low patients compared to *ERBB2*-high patients. This overexpression of the homologous recombination repair transcripts was associated with poor outcome in *ERBB2*-low tumors, not in *ERBB2*-high tumors. Furthermore, the combined expression of high *RAD21*/*RAD50*, high *RAD21*/*BARD1* or high *RAD50*/*BARD1* were significantly associated with worse DFS and a better outcome for those with low co-expression in the *ERBB2*-low cohort. High expression of either *RAD21*/*RAD50* or *RAD21*/*BARD1* in *ERBB2*-low cohort associated with higher chance of metastasis. In addition, gene expression of *BARD1* alone or in combination with *RAD50* acted as an independent prognostic factor for worst survival. The data presented in this study reveal a connection between *RAD21*, *RAD50*, *BARD1* and *ERBB2* and patient survival. Importantly, it provided novel findings and potential prognostic markers, particularly in *ERBB2*-low bladder cancer.

## Introduction

Bladder cancer, is the 10th most common type of cancer globally, with an estimated 600,000 diagnosed cases and over 200,000 deaths annually according to the latest GLOBOCAN report^1^. It remains as one of the most challenging cancers to diagnose, as diagnosis is mainly confirmed through an invasive procedure called cystoscopy^2,3^. Bladder cancer can be clinically stratified into a three-stage spectrum; non-muscle invasive bladder cancer (NMIBC), where the disease is affecting the inner layer of the bladder. Then, muscle-invasive bladder cancer (MIBC), where the bladder muscle tissue is affected as well. Finally, at the end of the spectrum is metastatic, which happens when the disease spread to the adjacent lymph nodes and other organs. Treatment for bladder cancer include surgery, radiation, chemotherapy, immunotherapy and targeted therapy^4^. Deciding on the best treatment course relay heavily on the clinical spectrum in which the case lies on, in addition to the associated molecular characteristics. One tool to characterize cancer cases is through biomarkers. A set of bladder cancer associated biomarkers are being investigated and assigned to different prognostic outcomes. These markers can be used to indicate disease metastasis or recurrence, as well as response to certain treatment^5^.

Human epidermal growth factor receptor 2 (HER2/*ERBB2*) is a member of the epithelial growth factor receptor family, a group of transmembrane receptor tyrosine kinases. This family of receptors play a role in cell proliferation, survival and mobility^6^. The overexpression of HER2 is known to be implicated in a number of malignancies, including breast and gastroesophageal cancers where HER2 targeted drugs are currently approved^7^. For bladder cancer, HER2 is still under investigation as a diagnostic, prognostic and targeted therapy approach^8^. A growing body of literature is reporting on the association between HER2 overexpression in bladder cancer and poor prognosis and clinical outcomes, in addition to the possible benefits of HER2-targeted therapies^9–12^. However, there are also contradictory reports on this matter owing to several factors including tumor grade and heterogeneity, as well as study methodology and patient selection^13–15^. This instigates the importance of further assessing HER2/*ERBB2* in the context of bladder cancer from different angles. This will allow for a better understanding of the over- and under-expression pattern, other linked biomarkers such as *RAD21*, *RAD50* and *BARD1*, which are investigated in this study, and effective targeting mechanisms.

*RAD21* is an essential gene that encodes a homologous recombination repair protein, this protein is a part of a multi-subunit cohesin complex (RAD21, SMC3, SMC1A and STAG1/2). Cohesin facilitates cohesion between replicated sister chromatids, plays a role in regulating gene expression and promotes accurate DNA repair through homologous recombination pathway. Just over a decade ago, cohesin mutations were found to be associated with cancer. Now, increasing evidence is showing that cohesin is in fact among the most commonly mutated protein complexes in cancers, including bladder cancer^16–19^. Of the four cohesin complex subunits; RAD21 is the most commonly overexpressed in cancers^20^. It was reported that RAD21 contributes to telomere maintenance, thus variants could lead to indefinite cell replication, which is a key characteristics in tumorigenicity^21^. RAD21 overexpression was implicated in different types of cancers including colorectal^22^, lung^23^, cervical^24^ ovarian^25^ prostate^26^ and breast cancer, where RAD21 overexpression was reported to confer poor prognosis and resistance to chemotherapy in HER2 mutant breast cancer patients^27^. It was also reported that RAD21 was overexpressed in bladder cancer tissues, it was proposed that RAD21 overexpression affected the RAD21 co-expressed cell cycle regulatory genes, which in turn affected cell cycle processes and contributed to tumorigenicity^28^.

RAD50 is a subunit of the MRN complex (MRE11/RAD50/NBS1), which plays a pivotal role in cellular response to DNA double-strand breakage by homologous recombination repair^29^. Defects in cellular responses to DNA damage instigate genome instability, which is a hallmark of cancer^30^. Mutations in the MRN complex, including *RAD50* is implicated in tumorigenicity. For example, mutation in *RAD50* was reported to be significantly associated with endometrioid endometrial carcinoma^31^. In breast cancer, *RAD50* mutations is not associated with increased risk, but it is associated with shorten survival^32^. Low RAD50 expression was also associated with poor survival in colorectal mucinous adenocarcinoma patients^33^ and in postoperative early stage/low-grade rectal cancer patients, as low RAD50 expression was associated with perineural invasion^34^. In outcome of radiotherapy investigation, low MRN complex expression is associated with high histologic grade and estrogen receptor negativity. In addition to indication of poor radiotherapy efficiency in early breast cancer patients^35^. Whereas another study that examined RAD50 reported that upregulation of RAD50 had the strongest correlation with radioresistance in lung cancer patients^36^. For bladder cancer, the expression of the MRE11 subunit was reported to predict radio therapy outcomes, as high expression was associated with better survival^37–39^, although the role of RAD50 is yet to be elucidated.

Breast cancer 1 gene (BRCA1) is one of the most implicated genes in hereditary breast and ovarian cancers. The *BARD1* gene is a BRCA1-associated RING domain 1 protein coding gene, which interacts with BRCA1 to repair damaged DNA^40^. Both of BARD1 and BRCA1 participates in homologous recombination repair pathway^41^. Therefore, mutations in *BARD1* as well as *BRCA1* will disrupt the BARD1-BRCA1 interaction, and therefore DNA damage repair. This instigates the importance of investigating *BARD1* mutations, especially in *BRCA1* mutation-negative cancers^42^. Furthermore, besides the BRCA1-dependant pathway, BARD1 was also found to play a role in tumor suppression via a BRCA1-independent pathways, such as the TP53-dependent pathway for apoptotic signaling^43^. Variants in the *BARD1* gene were linked to breast cancer as well^44–46^. *BARD1* variants were also linked to a number of non-breast, non-gynecological cancers^47^. For bladder cancer, there is not much literature on the matter. However, one recent study reported homologous recombination deficiency; one BARD1-deficient sample in three urothelial bladder tumor cohorts, compared to four BRCA2, three BRCA1 and two RBBP8-deficient samples^48^.

In the current study, we aimed to investigate the co-expression of *RAD21*, *RAD50* and *BARD1* mRNA levels in relation to *ERBB2* low and high expression. This is done to explore their impact on bladder cancer patient survival and cancer aggressiveness. Then, in order to identify the relationship, overlapping genes and functional enrichment pathways between our homologous recombination targets and *ERBB2* we constructed gene interaction network. Altogether, interpretation of the altered expression, prognostic and gene network relationship of our targets may reveal new insights into the prognostic knowledge of bladder cancer.

## Materials and methods

### Study cohorts and data analysis

In this retrospective study The Cancer Genome Atlas (TCGA) bladder cancer dataset was used as the main cohort along with four validation cohorts. *Cohort one*; TCGA datasets consist of 413 patients with MIBC and matched normal samples. Data were examined using UALCAN a publicly available interactive online portal (http://ualcan.path.uab.edu/index.html)^49^ and cBioPortal (https://www.cbioportal.org/) originally from Bladder Cancer (TCGA, Cell 2017). In this cohort, mRNA expression z-scores (RNA Seq V2 RSEM) were measured by Agilent microarray^50–52^. *Cohort two*; Memorial Sloan Kettering Cancer Center dataset (MSK, J Clin Onco 2013), this cohort consist of 97 high grade bladder urothelial carcinoma^53^. *Cohort three*; GSE31684 (Platform GPL570) from the Gene Expression Omnibus (GEO) database (https://www.ncbi.nlm.nih.gov/gds/). This cohort consist of 93 primary bladder cancer samples analyzed with Affymetrix Human Genome U133 Plus 2.0 Array. 68.82% of the patients in this cohort were over 65 years old and 31.18% where 65 year old or younger. Also, 73.12% where males vs. 26.88% female, with 93.55% of patients with high grade tumors and 6.45% with low grade. Tumor stages were as follow; 70.97% T4–T2 and 29.03% T1–Ta^54,55^. *Cohort four*; GSE48075 (Platform GPL6947) from GEO, consists of 142 primary bladder tumors (73 MIBC and 69 NMIBC) with tumor stages of 51.41% T4–T2 and 48.59% T1–Ta. Samples analyzed with Illumina HumanHT-12 V3.0 expression beadchip^56,57^. *Cohort five*; E-MTAB-4321 from ArrayExpress (https://www.ebi.ac.uk/biostudies/arrayexpress) which consists of 476 primarily early-stage urothelial carcinoma (460 NMIBC), samples analyzed by sequencing assay. 64.50% of the patients in this cohort were over 65 years old and 35.50% where 65 years old or younger. In addition, 77.10% of the whole cohort were male and 22.90% were female. Low grade tumors constituted 58.19% whereas high grade were 40.34%. Tumor stages of the whole cohort were as follow; 96.64% of patients Ta–T1 and 3.36% T2–T4^58^.

The different expressions of *RAD21*, *RAD50* and *BARD1* across all five cohorts were investigated based on the median cut-off point of *ERBB2* data of each cohort. Therefore, patients with *ERBB2* expression values lower than the median cut-off point were considered as *ERBB2*-low patients. Then the total gene expression of each targeted genes was investigated further in these sub-cohorts of *ERBB2* low and high.

### Gene–gene interaction network construction and analysis

For gene–gene interaction network between *RAD21*, *RAD50*, *BARD1* and *ERBB2* we used the GeneMANIA Cytoscape plugin (https://apps.cytoscape.org/apps/genemania)^59,60^. Interaction network covering; physical interactions, co-expression, co-localization, genetic interactions, pathway and shared protein domains. With max 100 genes interaction and max attributes. Network structure was visualized by Cytoscape (https://cytoscape.org/)^61^. To further analyze and calculate the topology parameters (Node degrees, Betweenness centrality and Closeness centrality) of the network, NetworkAnalyzer^62^, a plugin in Cytoscape, was applied.

### Gene ontology (GO) functional and pathway enrichment analysis

To provide Gene Ontology analysis we used the Database for Annotation, Visualization and Integrated Discovery tool (DAVID; latest version Dec. 2021: https://david.ncifcrf.gov/home.jsp). This tool includes biological process, molecular function, cellular component and also Kyoto Encyclopedia of Gene and Genomes (KEGG) pathway analysis^63^. Enrichment analysis was performed with the threshold of *p* < 0.05.

### Statistical analyses

Data analysis were performed using JMP Pro 15 (SAS Institute Inc., USA). For the prognostic significance survival curves, Kaplan–Meier method was used with log-rank comparison for significance testing. In the univariate analysis, Chi-square test (χ^2^) was used to evaluate the relationship between *RAD21*, *RAD50*, *BARD1* and *ERBB2* expression and clinicopathological variables. In multivariate analysis, to emphasize on *RAD21*, *RAD50*, *BARD1* and *ERBB2* interaction, a Cox proportional hazard model was used for the multivariate survival analysis including all potential confounder factors. The proportional hazards assumption was checked, the relationship between log cumulative hazard and a covariate was linear. Where appropriate, two-tailed Student’s t-test was performed using GraphPad Prism (version 9.5.0, USA). All differences were considered statistically significant at *p* < 0.05, *p* values were two-sided; all confidence intervals were at 95%.

## Results

### Expression of *ERBB2*, *RAD21*, *RAD50* and *BARD1* in bladder tissues

**Figure 1:**
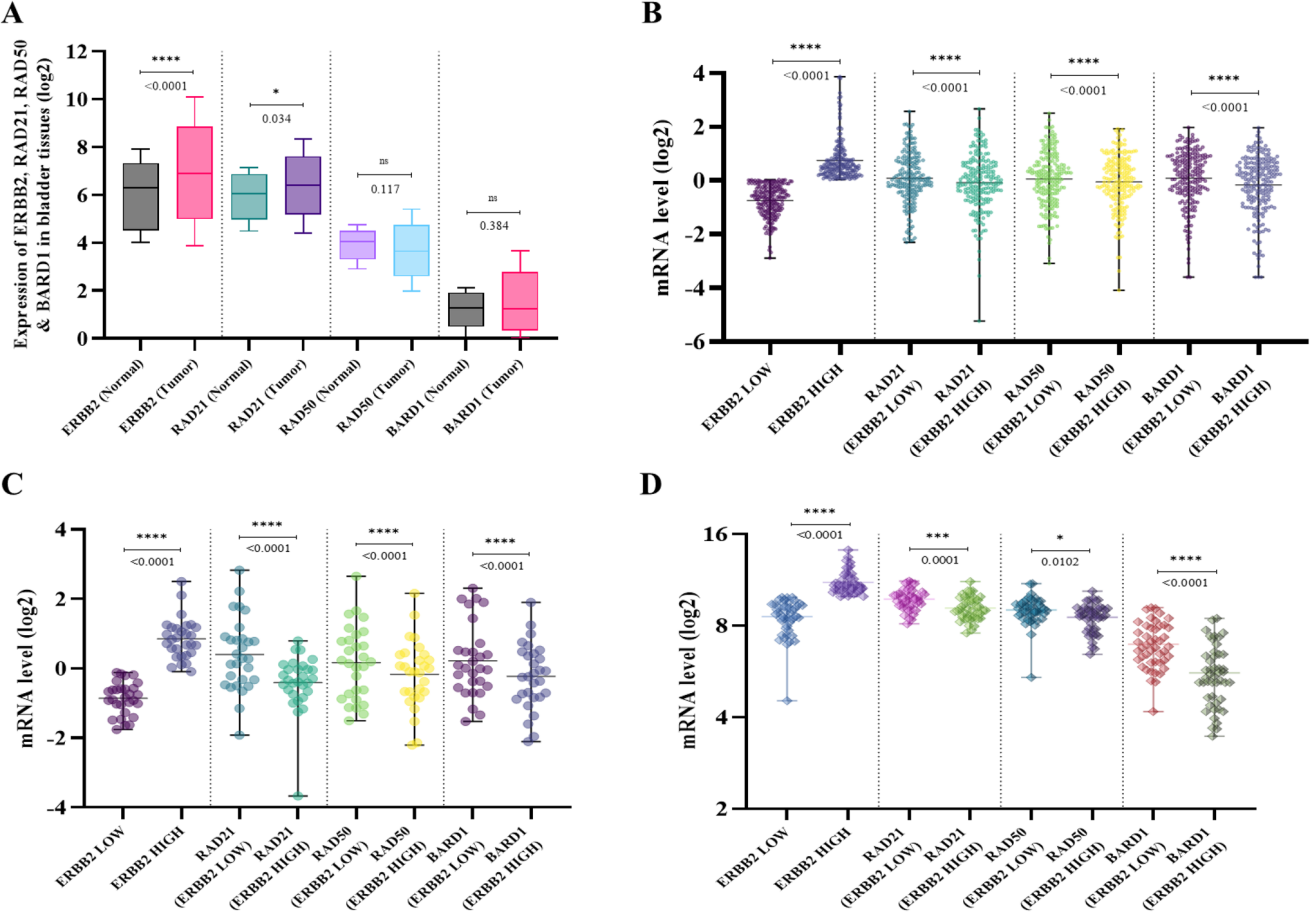
(**A**) Boxplot of the mRNA expression levels of *ERBB2*, *RAD21*, *RAD50* and *BARD1* in bladder cancer tissue, along with matching normal tissue. (**B**) TCGA dot plot showing the mRNA expression levels of *ERBB2* in bladder cancer patients, and *RAD21*, *RAD50* and *BARD1* expressions at different *ERBB2* levels. (**C**) MSK dot plot showing the mRNA expression levels of *ERBB2* in bladder cancer patients, and *RAD21*, *RAD50* and *BARD1* expressions at different *ERBB2* levels. (**D**) GEO-GSE31684 dot plot showing the mRNA expression levels of *ERBB2* in bladder cancer patients, and *RAD21*, *RAD50* and *BARD1* expressions at different *ERBB2* levels. *p < 0.05, **p < 0.01, ***p < 0.001 and ****p < 0.0001. All data were analyzed using the two-tailed Student’s t-test.

We initially compared the total expression levels of *ERBB2*, *RAD21*, *RAD50* and *BARD1* mRNA in normal and tumor bladder tissues with bioinformatics analyses using the TCGA database (*Cohort one*). The cohort consists of 413 patients with MIBC and matched normal samples, the TCGA datasets were previously described^64^. The data revealed a significantly high mRNA expression levels of *ERBB2* and *RAD21* in tumor tissues compared to normal; median = 6.888 tumor vs. 6.299 normal; p < 0.0001 and median = 6.408 tumor vs. 6.043 normal; p = 0.034, Fig. 1A respectively. *RAD50* and *BARD1* mRNA levels showed no significant difference between tumors and the respective normal tissues (Fig. 1A). Interestingly, when we sub grouped patients according to *ERBB2* status (*ERBB2*-low and *ERBB2*-high), we found that *RAD21*, *RAD50* and *BARD1* expression levels increased significantly in *ERBB2*-low patients compared to *ERBB2*-high patients. Figure 1B, shows *RAD21* expression median = 0.02 in *ERBB2-*low compared to *RAD21* median = − 0.07 in *ERBB2-*high; p < 0.0001. *RAD50* expression median = 0.12 in *ERBB2-*low compared to *RAD50* median = − 0.02 in *ERBB2-*high; p < 0.0001. *BARD1* expression median = 0.21 in *ERBB2-*low compared to *BARD1* median = 0.08 in *ERBB2-*high; p < 0.0001. We validated this finding with *Cohort two* from MSK dataset (Fig. 1C). As expected, total *RAD21* in *ERBB2*-low cohort increased significantly to the same in *ERBB2*-high cohort (median = 0.29 vs. median = − 0.34; p < 0.0001). Total *RAD50* expression in *ERBB2-*low patients was higher compared to *RAD50* in *ERBB2*-high cohort (median = 0.20 vs. median = − 0.09; p < 0.0001). The same significant trend was shown with *BARD1* expression in different *ERBB2* status (median = 0.01 vs. median = − 0.24; p < 0.0001). The second validation dataset (Fig. 1D), *Cohort three* from GEO-GSE31684 confirmed the elevated expression of the three homologous recombination mRNAs in different *ERBB2* status. Data confirmed significant increase of *RAD21, RAD50* and *BARD1* expression in *ERBB2*-low patients compared to *ERBB2*-high patients as follow: total *RAD21* (median = 9.81 vs. median = 9.00; p = 0.0001), total *RAD50* (median = 9.00 vs. median = 8.70; p = 0.0102) and total *BARD1* (median = 6.81 vs. median = 5.45; p < 0.0001).

### Association between homologous recombination repair transcripts (*RAD21*, *RAD50* and *BARD1*) with *ERBB2* and survival

**Figure 2:**
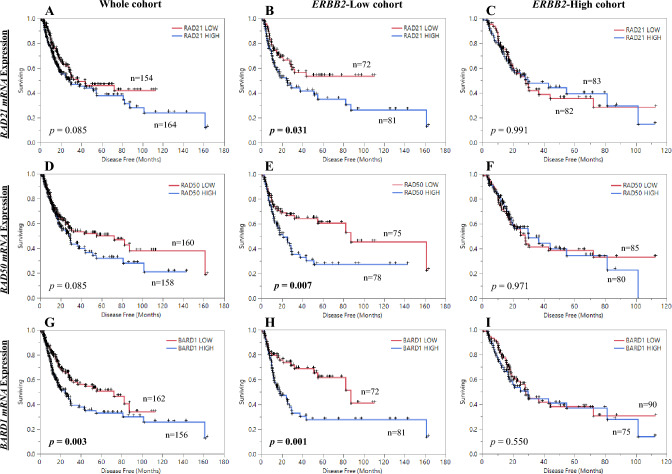
Kaplan–Meier analysis for bladder cancer data; Disease free survival (DFS) of *RAD21* mRNA expression in (**A**) whole cohort, (**B**) *ERBB2*-Low cohort, (**C**) *ERBB2*-High cohort. DFS of *RAD50* mRNA expression in (**D**) whole cohort, (**E**) *ERBB2*-Low cohort, (**F**) *ERBB2*-High cohort. DFS of *BARD1* mRNA expression in (**G**) whole cohort, (**H**) *ERBB2*-Low cohort, (**I**) *ERBB2*-High cohort.

The potential prognostic value of *RAD21*, *RAD50* and *BARD1* mRNAs expression in different *ERBB2* status was assessed using the largest bladder TCGA dataset (*Cohort one*). In the whole cohort *RAD21* expression alone did not influence survival on the disease-free survival (DFS) (p = 0.085; Fig. 2A) and in *ERBB2*-high cohort (p = 0.991; Fig. 2C). High *RAD21* mRNA was significantly associated with poor survival in the *ERBB2*-low cohort (p = 0.031; Fig. 2B). Poor survival of high *RAD21* mRNA was also associated with *ERBB2*-low cohort of the overall survival (OS), with 5-year OS rate of 34.7% high vs. 40.4% with low *RAD21,* though not significant (Additional file 1: Fig. S1A–C). In additional cohorts of bladder cancer, patients with high *RAD21* mRNA in the *ERBB2*-low cohorts showed tendency toward poor survival [*Cohorts*: *Cohort four* (GEO-GSE48075; with 5-year survival rates of 29.9% high vs. 53.7% low *RAD21*) and *Cohort five* the NMIBC (E-MTAB-4321; with 5-years survival rates of 83.3% high vs. 91.4% low *RAD21*)]; (Additional file 1: Fig. S2A,B, respectively). *RAD50* transcript level (*Cohort one*) did not influence survival in the whole cohort (p = 0.085; Fig. 2D) and in *ERBB2*-high cohort (p = 0.971; Fig. 2F). Though high *RAD50* mRNA was significantly associated with poor survival in the *ERBB2*-low cohort (p = 0.007; Fig. 2E). High *RAD50* mRNA expression also showed tendency toward poor survival in the *ERBB2*-low cohort of the OS, with 5-year OS rate of 30.5% high vs. 44.6% with low *RAD50,* though not significant (Additional file 1: Fig. S1D–F). This finding was validated in *Cohort three*, showing poor recurrence free survival for patients with high *RAD50* mRNA in *ERBB2*-low cohort (5-year survival rate of 38.7% high *RAD50* vs. 71.4% low *RAD50*) compared to the whole cohort, or *ERBB2*-high cohort (Additional file 1: Fig. S3A). High *BARD1* mRNA was significantly associated with poor DFS in the whole cohort (p = 0.003; Fig. 2G) and in *ERBB2*-low cohort (p = 0.001; Fig. 2H), but not in *ERBB2*-high cohort (p = 0.550; Fig. 2I). The same tendency toward poor survival was detected in *Cohort one* (5-year OS rate of 31.1% high vs. 45.0% with low *BARD1*) and *Cohort four* between high *BARD1* and low *ERBB2* patients, with 5-year OS rate of 23.5% high vs. 49.9% with low *BARD1* (Additional file 1: Figs. S1G–I and S3B).

**Figure 3:**
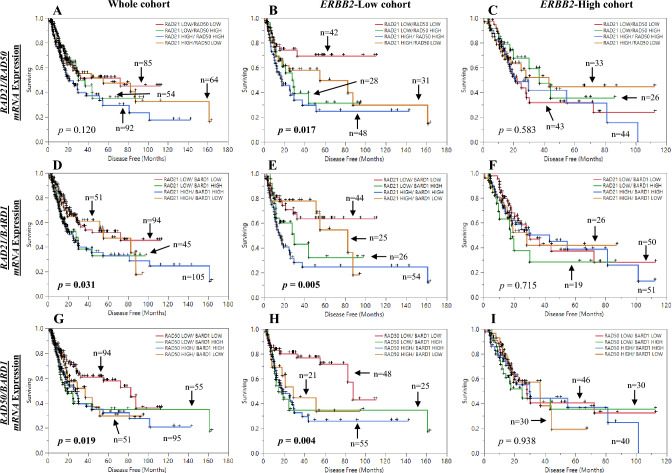
Kaplan–Meier analysis of bladder cancer data; Disease free survival (DFS) of *RAD21/RAD50* mRNA expression in (**A**) whole cohort, (**B**) *ERBB2*-Low cohort, (**C**) *ERBB2*-High cohort. DFS of *RAD21/BARD1* mRNA expression in (**D**) whole cohort, (**E**) *ERBB2*-Low cohort, (**F**) *ERBB2*-High cohort. DFS of *RAD50/BARD1* mRNA expression in (**G**) whole cohort, (**H**) *ERBB2*-Low cohort, (**I**) *ERBB2*-High cohort.

Furthermore, investigating the homologous recombination repair transcripts (*RAD21*, *RAD50* and *BARD1*) with each other revealed that combined expression of high *RAD21*/high *RAD50* significantly associated with worst DFS and better outcome for those with low *RAD21*/low *RAD50* in the *ERBB2*-low cohort (p = 0.017; Fig. 3B). No significant different in the whole cohort and in the *ERBB2*-high cohort (Fig. 3A,C). Data also showed a tendency toward poor OS with high *RAD21*/high *RAD50* (5-year OS of 29.5%) and better with low *RAD21*/low *RAD50* (5-year OS of 45.4%) in *ERBB2*-low patients, but the trend was not significant (Additional file 1: Fig. S4A–C). Similarly, combined high *RAD21*/high *BARD1* associated significantly with worst outcome in the whole cohort and *ERBB2*-low cohort (p = 0.031, p = 0.005; Fig. 3D,E; respectively). Whereas, no significant association was found in the *ERBB2*-high patients (Fig. 3F). High *RAD21*/high *BARD1* showed a tendency toward poor OS with 5-year of 29.2% vs. 42.5% with low *RAD21*/low *BARD1* in *ERBB2*-low cohort (Additional file 1: Fig. S4D–F). Then again, low *RAD50/* low *BARD1* mRNA expression showed a significantly better DFS compared to other subgroups in the whole cohort and in the *ERBB2*-low cohort (p = 0.019, p = 0.004; Fig. 3G,H; respectively). The OS was also better with 51.5% 5-year rate vs. 31.1% with high *RAD50*/high *BARD1,* though not significant (Additional file 1: Fig. S4G,H). Finally, no significant DFS and OS differences were found in any group among the *ERBB2*-high cohort (Fig. 3I and Additional file 1: Fig. S4I).

### *RAD21*,* RAD50* and *BARD1* mRNA levels and clinicopathological features

To further evaluate the impact of *RAD21*, *RAD50* or *BARD1* mRNAs with *ERBB2* status on the clinicopathological variables, we used the TCGA database (*Cohort one*). We previously described the *ERBB2* distribution of the clinicopathological characteristics of this cohort^64^. Univariate analysis data indicate that in *ERBB2*-high cohort mRNA expression of *RAD21* low was significantly associated with tumor grade (p = 0.011). Also, *BARD1* low was significantly associated with tumor grade (p = 0.04) and non-papillary tumor shape (p = 0.037). However, no association was observed in *ERBB2*-low cohort (Table 1).

**Table 1:** Association of RAD21, RAD50 and BARD1 mRNAs expression and clinicopathological variables in TCGA dataset.

		*ERBB2*-low cohort															*ERBB2*-high cohort														
		*RAD21* LOW		*RAD21 HIGH*		*p* value	*RAD50* LOW		*RAD50 HIGH*		*p* value	*BARD1* LOW		*BARD1 HIGH*		*p* value	*RAD21* LOW		*RAD21 HIGH*		*p* value	*RAD50* LOW		*RAD50 HIGH*		*p* value	*BARD1* LOW		*BARD1 HIGH*		*p* value
		N	%	N	*%*		N	%	N	*%*		N	%	N	%		N	%	N	*%*		N	%	N	*%*		N	%	N	%	
Group age (years)	≤ 65	38	46.30	44	53.70	0.69	34	41.50	48	58.50	0.15	38	46.30	44	53.70	0.87	46	58.20	33	41.80	0.15	40	50.60	39	49.40	0.68	44	55.70	35	44.30	0.53
	> 65	60	49.20	62	50.80		63	51.60	59	48.40		58	47.50	64	52.50		60	48.00	65	52.00		67	53.60	58	46.40		64	51.20	61	48.80	
	Unknown	0	0.00	0	0.00		0	0.00	0	0.00		0	0.00	0	0.00		0	0.00	0	0.00		0	0.00	0	0.00		0	0.00	0	0.00	
Gender	Male	66	45.80	78	54.20	0.28	72	50.00	72	50.00	0.32	68	47.20	76	52.80	0.98	79	50.60	77	49.40	0.50	79	50.60	77	49.40	0.35	79	50.60	77	49.40	0.24
	Female	32	54.20	27	45.80		25	42.40	34	57.60		28	47.50	31	52.50		27	56.30	21	43.80		28	58.30	20	41.70		29	60.40	19	39.60	
	Unknown	0	0.00	1	100.00		0	0.00	1	100.00		0	0.00	1	100.00		0	0.00	0	0.00		0	0.00	0	0.00		0	0.00	0	0.00	
Tumor grade	Low grade	2	50.00	2	50.00	0.94	2	50.00	2	50.00	0.93	3	75.00	1	25.00	0.26	14	82.40	3	17.60	.011*	9	52.90	8	47.10	0.98	13	76.50	4	23.50	0.04*
	High grade	96	48.20	103	51.80		95	47.70	104	52.30		93	46.70	106	53.30		92	50.00	92	50.00		98	53.30	86	46.70		94	51.10	90	48.90	
	Unknown	0	0.00	1	100.00		0	0.00	1	100.00		0	0.00	1	100.00		0	0.00	3	100.00		0	0.00	3	100.00		1	33.33	2	66.67	
Tumor stage	TX	0	0.00	1	100.00	0.50	0	0.00	1	100.00	0.70	1	100.00	0	0.00	0.37	0	0.00	0	0.00	0.09	0	0.00	0	0.00	0.16	0	0.00	0	0.00	0.22
	T0	0	0.00	0	0.00		0	0.00	0	0.00		0	0.00	0	0.00		0	0.00	1	100.00		0	0.00	1	100.00		0	0.00	1	100.00	
	T1	0	0.00	2	100.00		0	0.00	2	100.00		1	50.00	1	50.00		1	100.00	0	0.00		0	0.00	1	100.00		1	100.00	0	0.00	
	T2	9	64.30	5	35.70		9	64.30	5	35.70		11	78.60	3	21.40		15	62.50	9	37.50		15	62.50	9	37.50		17	70.80	7	29.20	
	T2a	3	42.90	4	57.10		4	57.10	3	42.90		4	57.10	3	42.90		6	33.30	12	66.70		8	44.40	10	55.60		6	33.30	12	66.70	
	T2b	17	53.10	15	46.90		15	46.90	17	53.10		13	40.60	19	59.40		10	41.70	14	58.30		14	58.30	10	41.70		12	50.00	12	50.00	
	T3	7	33.30	14	66.70		8	38.10	13	61.90		6	28.60	15	71.40		15	71.40	6	28.60		6	40.00	9	60.00		10	47.60	11	52.40	
	T3a	12	35.30	22	64.70		17	50.00	17	50.00		16	47.10	18	52.90		21	58.30	15	41.70		8	38.10	13	61.90		19	52.80	17	47.20	
	T3b	25	53.20	22	46.80		22	46.80	25	53.20		23	48.90	24	51.10		18	52.90	16	47.10		20	55.60	16	44.40		21	61.80	13	38.20	
	T4	3	42.90	4	57.10		3	42.90	4	57.10		4	57.10	3	42.90		3	100.00	0	0.00		23	67.60	11	32.40		1	33.30	2	66.70	
	T4a	10	52.60	9	47.40		9	47.40	10	52.60		10	52.60	9	47.40		12	50.00	12	50.00		3	100.00	0	0.00		12	50.00	12	50.00	
	T4b	1	50.00	1	50.00		0	0.00	2	100.00		1	50.00	1	50.00		0	0.00	3	100.00		9	37.50	15	62.50		0	0.00	3	100.00	
	Unknown	11	61.11	7	38.89		10	55.56	8	44.44		0	0.00	0	0.00		5	33.33	10	66.67		1	33.30	2	66.70		9	60.00	6	40.00	
Disease stage	Stage I	0	0.00	1	100.00	0.70	0	0.00	1	100.00	0.27	0	0.00	1	100.00	0.51	1	100.00	0	0.00	0.52	0	0.00	1	100.00	0.77	1	100.00	0	0.00	0.22
	Stage II	31	52.50	28	47.50		34	57.60	25	42.40		30	50.80	29	49.20		34	47.90	37	52.10		38	53.50	33	46.50		40	56.30	31	43.70	
	Stage III	40	47.10	45	52.90		37	43.50	48	56.50		36	42.40	49	57.60		32	58.20	23	41.80		29	52.70	26	47.30		33	60.00	22	40.00	
	Stage IV	27	47.40	30	52.60		26	45.60	31	54.40		29	50.90	28	49.10		39	51.30	37	48.70		40	52.60	36	47.40		34	44.70	42	55.30	
	Unknown	0	0.00	2	100.00		0	0.00	2	100.00		1	50.00	1	50.00		0	0.00	1	100.00		0	0.00	1	100.00		0	0.00	1	100.00	
Tumor shape	Papillary	75	49.70	76	50.30	0.83	73	48.30	78	51.70	0.76	68	45.00	83	55.00	0.11	56	47.10	63	52.90	0.08	64	53.80	55	46.20	0.60	56	47.10	63	52.90	.037*
	Non-papillary	23	47.90	25	52.10		22	45.80	26	54.20		28	58.30	20	41.70		50	59.50	34	40.50		42	50.00	42	50.00		52	61.90	32	38.10	
	Unknown	0	0.00	5	100.00		2	40.00	3	60.00		0	0.00	5	100.00		0	0.00	1	100.00		1	100.00	0	0.00		0	0.00	1	100.00	
Lymph node	No	18	54.50	15	45.50	0.61	16	48.50	17	51.50	0.93	18	54.50	15	45.50	0.43	29	63.00	17	37.00	0.05	25	54.30	21	45.70	0.79	28	60.90	18	39.10	0.13
	Yes	74	49.70	75	50.30		71	47.70	78	52.30		70	47.00	79	53.00		66	46.50	76	53.50		74	52.10	68	47.90		68	47.90	74	52.10	
	Unknown	6	27.27	16	72.72		10	45.45	12	54.55		8	36.36	14	63.64		11	68.75	5	31.25		8	50.00	8	50.00		12	75.00	4	25.00	
Metastasis	No	37	42.50	50	57.50	0.16	35	40.20	52	59.80	0.06	36	41.40	51	58.60	0.14	55	50.90	53	49.10	0.70	53	49.10	55	50.90	0.27	56	51.90	52	48.10	0.68
	Yes	60	52.60	54	47.40		61	53.50	53	46.50		59	51.80	55	48.20		51	53.70	44	46.30		54	56.80	41	43.20		52	54.70	43	45.30	
	Unknown	1	33.33	2	66.67		1	33.33	2	66.67		1	33.33	2	66.67		0	0.00	1	100.00		0	0.00	1	100.00		0	0.00	1	100.00	

Table 2 summarizes the association between the co-expression of the homologous recombination repair transcripts with *ERBB2* and the clinicopathological features. Analyzing the combined high expression of either *RAD21*/*RAD50* or *RAD21*/*BARD1* in *ERBB2*-low cohort had a significant association with higher chance of metastasis (p = 0.011). On the other hand, low expression of *RAD50/BARD1* in *ERBB2*-low cohort had a significant association with higher tumor stages (p = 0.013). The high expression of *RAD50/BARD1* correlated significantly with papillary tumor shape (p = 0.035) (Table 2). No significant association with any co-expression was observed in *ERBB2*-high cohort (Additional file 2: Table S1).

**Table 2:** Clinicopathological significance of the combined RAD21, RAD50 and BARD1 mRNAs expression in low ERBB2 cohort.

		*ERBB2*-low cohort																													
		*RAD21* LOW/RAD50 LOW		RAD21 LOW/RAD50 HIGH		*p* value	*RAD21* HIGH/RAD50 LOW		RAD21 HIGH/RAD50 HIGH		*p* value	*RAD21* LOW/BARD1 LOW		RAD21 LOW/BARD1 HIGH		*p* value	*RAD21* HIGH/BARD1 LOW		RAD21 HIGH/BARD1 HIGH		*p* value	*RAD50* LOW/BARD1 LOW		RAD50 LOW/BARD1 HIGH		*p* value	*RAD50* HIGH/BARD1 LOW		RAD50 HIGH/BARD1 HIGH		*p* value
		N	%	N	*%*		N	%	N	*%*		N	%	N	*%*		N	%	N	*%*		N	%	N	*%*		N	%	N	*%*	
Group age (years)	≤ 65	18	47.40	20	52.60	0.04*	28	63.60	16	36.40	0.93	24	63.20	14	36.80	0.72	14	31.80	30	68.20	0.76	23	67.60	11	32.40	0.92	15	31.30	33	68.80	0.64
	> 65	41	68.30	19	31.70		40	64.50	22	35.50		40	66.70	20	33.30		18	29.00	44	71.00		42	66.70	21	33.30		16	27.10	43	72.90	
	Unknown	0	0.00	0	0.00		0	0.00	0	0.00		0	0.00	0	0.00		0	0.00	0	0.00		0	0.00	0	0.00		0	0.00	0	0.00	
Gender	Male	45	68.18	21	31.82	0.020*	27	34.62	51	65.38	0.57	44	66.67	22	33.33	0.68	24	30.77	54	69.23	0.91	49	68.06	23	31.94	0.71	19	26.39	53	73.61	0.35
	Female	14	43.75	18	56.25		11	40.74	16	59.26		20	62.50	12	37.50		8	29.63	19	70.37		16	64.00	9	36.00		12	35.29	22	64.71	
	Unknown	0	0.00	0	0.00		0	0.00	1	100.00		0	0.00	0	0.00		0	0.00	1	100.00		0	0.00	0	0.00		0	0.00	1	100.00	
Tumor grade	Low Grade	1	50.00	1	50.00	0.77	1	50.00	1	50.00	0.68	2	100.00	0	0.00	0.30	1	50.00	1	50.00	0.55	2	100.00	0	0.00	0.32	1	50.00	1	50.00	0.52
	High Grade	58	60.42	38	39.58		37	35.92	66	64.08		62	64.58	34	35.42		31	30.10	72	69.90		63	66.32	32	33.68		30	28.85	74	71.15	
	Unknown	0	0.00	0	0.00		0	0.00	1	100.00		0	0.00	0	0.00		0	0.00	1	100.00		0	0.00	0	0.00		0	0.00	1	100.00	
Tumor stage	TX	0	0.00	0	0.00	0.51	0	0.00	1	100.00	0.92	0	0.00	0	0.00	0.23	1	100.00	0	0.00	0.68	0	0.00	0	0.00	0.013*	1	100.00	0	0.00	0.64
	T0	0	0.00	0	0.00		0	0.00	0	0.00		0	0.00	0	0.00		0	0.00	0	0.00		0	0.00	0	0.00		0	0.00	0	0.00	
	T1	0	0.00	0	0.00		0	0.00	2	100.00		0	0.00	0	0.00		1	50.00	1	50.00		0	0.00	0	0.00		1	50.00	1	50.00	
	T2	7	77.78	2	22.22		2	40.00	3	60.00		9	100.00	0	0.00		2	40.00	3	60.00		9	100.00	0	0.00		2	40.00	3	60.00	
	T2a	3	100.00	0	0.00		1	25.00	3	75.00		3	100.00	0	0.00		1	25.00	3	75.00		4	100.00	0	0.00		0	0.00	3	100.00	
	T2b	8	47.06	9	52.94		7	46.67	8	53.33		8	47.06	9	52.94		5	33.33	10	66.67		9	60.00	6	40.00		4	23.53	13	76.47	
	T3	4	57.14	3	42.86		4	28.57	10	71.43		4	57.14	3	42.86		2	14.29	12	85.71		2	25.00	6	75.00		4	30.77	9	69.23	
	T3a	8	66.67	4	33.33		9	40.91	13	59.09		7	58.33	5	41.67		9	40.91	13	59.09		9	52.94	8	47.06		7	41.18	10	58.82	
	T3b	15	60.00	10	40.00		7	31.82	15	68.18		18	72.00	7	28.00		5	22.73	17	77.27		18	81.82	4	18.18		5	20.00	20	80.00	
	T4	1	33.33	2	66.67		2	50.00	2	50.00		2	66.67	1	33.33		2	50.00	2	50.00		2	66.67	1	33.33		2	50.00	2	50.00	
	T4a	5	50.00	5	50.00		4	44.44	5	55.56		7	70.00	3	30.00		3	33.33	6	66.67		7	77.78	2	22.22		3	30.00	7	70.00	
	T4b	0	0.00	1	100.00		0	0.00	1	100.00		1	100.00	0	0.00		0	0.00	1	100.00		0	0.00	0	0.00		1	50.00	1	50.00	
	Unknown	8	72.72	3	27.27		2	28.57	5	71.43		5	45.45	6	54.55		1	14.29	6	85.71		5	50.00	5	50.00		1	12.50	7	87.50	
Disease stage	Stage I	0	0.00	0	0.00	0.15	0	0.00	1	100.00	0.80	0	0.00	0	0.00	0.93	0	0.00	1	100.00	0.36	0	0.00	0	0.00	0.44	0	0.00	1	100.00	0.57
	Stage II	23	74.19	8	25.81		11	39.29	17	60.71		21	67.74	10	32.26		9	32.14	19	67.86		25	73.53	9	26.47		5	20.00	20	80.00	
	Stage III	22	55.00	18	45.00		15	33.33	30	66.67		26	65.00	14	35.00		10	22.22	35	77.78		22	59.46	15	40.54		14	29.17	34	70.83	
	Stage IV	14	51.85	13	48.15		12	40.00	18	60.00		17	62.96	10	37.04		12	40.00	18	60.00		18	69.23	8	30.77		11	35.48	20	64.52	
	Unknown	0	0.00	0	0.00		0	0.00	2	100.00		0	0.00	0	0.00		1	50.00	1	50.00		0	0.00	0	0.00		0	0.00	1	100.00	
Tumor shape	Papillary	46	61.33	29	38.67	0.68	27	35.53	49	64.47	0.97	47	62.67	28	37.33	0.32	21	27.63	55	72.37	0.13	49	67.12	24	32.88	0.62	19	24.36	59	75.64	0.035*
	Non-Papillary	13	56.52	10	43.48		9	36.00	16	64.00		17	73.91	6	26.09		11	44.00	14	56.00		16	72.73	6	27.27		12	46.15	14	53.85	
	Unknown	0	0.00	0	0.00		2	40.00	3	60.00		0	0.00	0	0.00		0	0.00	5	100.00		0	0.00	2	100.00		0	0.00	3	100.00	
Lymph node	No	12	66.67	6	33.33	0.51	4	26.67	11	73.33	0.43	13	72.22	5	27.78	0.42	5	33.33	10	66.67	0.92	13	81.25	3	18.75	0.24	5	29.41	12	70.59	1.00
	Yes	43	58.11	31	41.89		28	37.33	47	62.67		46	62.16	28	37.84		24	32.00	51	68.00		47	66.20	24	33.80		23	29.49	55	70.51	
	Unknown	4	66.67	2	33.33		6	37.50	10	62.50		5	83.33	1	16.67		3	18.75	13	81.25		5	50.00	5	50.00		3	25.00	9	75.00	
Metastasis	No	23	62.16	14	37.84	0.71	12	24.00	38	76.00	0.011*	27	72.97	10	27.03	0.25	9	18.00	41	82.00	0.011*	24	68.57	11	31.43	0.89	12	23.08	40	76.92	0.22
	Yes	35	58.33	25	41.67		26	48.15	28	51.85		37	61.67	23	38.33		22	40.74	32	59.26		41	67.21	20	32.79		18	33.96	35	66.04	
	Unknown	1	100.00	0	0.00		0	0.00	2	100.00		0	0.00	1	100.00		1	50.00	1	50.00		0	0.00	1	100.00		1	50.00	1	50.00	

Significant values are in bold.

Multivariate analysis of *RAD21*, *RAD50* or *BARD1* mRNAs expression alone or in combination was conducted. This was done to investigate whether the expressions are an independent prognostic factor. As shown in Table 3, multivariate analyses of the above factors together with tumor stage were conducted. *BARD1* mRNAs expression was an independent prognostic factor for worse DFS in the *ERBB2*-low cohort (p = 0.047, Hazard ratio 1.812, 95% CI 1.009–3.330), but not in *ERBB2*-high cohort. Similarly, in the *ERBB2*-low cohort combination of *RAD50*/*BARD1* mRNA expression was an independent factor for poor DFS (p = 0.008, Hazard ratio 1.378, 95% CI 1.088–1.760). Whereas tumor stage was an independent prognostic factor for poor DFS in *ERBB2*-high cohort (< 0.001, Hazard ratio 1.295, 95% CI 1.154–1.458).

**Table 3:** Multivariate analysis for predictors of disease free survival.

Factors	Disease free survival							
	ERBB2-low cohort				ERBB2-high cohort			
	Hazard ratio	95% Confidence interval			Hazard ratio	95% Confidence interval		
		Lower bound	Upper bound	p value		Lower bound	Upper bound	p value
*RAD21* expression	1.355	0.823	2.276	0.235	1.010	0.597	1.713	0.971
*RAD50* expression	1.363	0.790	2.403	0.269	0.905	0.555	1.465	0.685
*BARD1* expression	1.812	1.009	3.330	**0.047**	1.171	0.683	2.010	0.566
*RAD21*/*RAD50* expression	1.224	0.746	2.003	0.423	0.726	0.480	1.097	0.128
*RAD21*/*BARD1* expression	1.007	0.637	1.589	0.975	1.359	0.892	2.070	0.152
*RAD50*/*BARD1* expression	1.378	1.088	1.760	**0.008**	1.003	0.801	1.253	0.979
Tumor Stage	1.104	0.972	1.257	0.128	1.295	1.154	1.458	**< 0.0001**

*ERBB2* Erb-B2 Receptor Tyrosine Kinase 2, *RAD21* RAD21 Cohesin Complex Component, *RAD50* RAD50 Double Strand Break Repair, *BARD1* BRCA1 Associated RING Domain 1, RAD21/RAD50, RAD21/BARD1, RAD50/BARD1, co-expression. Hazard ratio, 95% Confidence Interval and p-value are shown. Significant results are highlighted in bold.

### Gene interaction network of *RAD21*, *RAD50, BARD1 *and *ERBB2*

**Figure 4:**
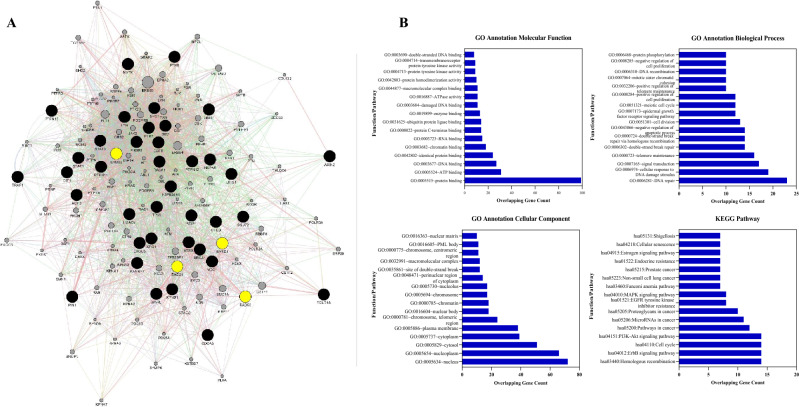
(**A**) Gene–Gene interaction network demonstrating 100 overlapping genes, along with *ERBB2*, *RAD21*, *RAD50* and *BARD1*. (**B**) GO functional enrichment and KEGG pathway analyses of the all-overlapping genes, *ERBB2*, *RAD21*, *RAD50* and *BARD1*. The top significant enriched GO annotation Molecular Function, Biological Process, Cellular Component, KEGG pathway analyses.

A gene interaction network was constructed for the three homologous recombination repair transcripts (*RAD21*, *RAD50* and *BARD1*) and *ERBB2*. This was done to identify the most related genes network between our targets. The network was constructed using the GeneMANIA Cytoscape plugin^59^. Our network was based on the top 100 genes showing 104 nodes and 2239 interactions (Fig. 4A and Additional file 2: Table S2). Interaction percentages in the network were: 82.19% physical interactions, 8.40% co-expression, 3.78% co-localization, 2.99% genetic interactions, 2.01% pathway and 0.64% shared protein domains. In addition, a network interaction was analyzed illustrating the node degrees, betweenness centrality and closeness centrality with Network analyzer of the 100 top genes, as shown in Table 4.

**Table 4:** ERBB2, RAD21, RAD50 and BARD1 Gene network analyzed by Cytoscape Networkanalyzer Tool. Top 100 gene with high betweenness centrality, closeness centrality and degree.

	Gene name	Betweenness centrality	Closeness centrality	Degree		Gene name	Betweenness centrality	Closeness centrality	Degree		Gene name	Betweenness centrality	Closeness centrality	Degree		Gene name	Betweenness centrality	Closeness centrality	Degree
1	*BRCA1*	0.034370043	0.725352113	117	27	*ERBB4*	0.021614369	0.624242424	60	53	*TGFA*	0.00994864	0.578651685	37	79	*NRG2*	0.001453903	0.528205128	23
2	*BARD1*	0.015171864	0.651898734	102	28	*RAD51*	0.002041194	0.575418994	59	54	*PLCG2*	0.003618275	0.54787234	35	80	*UBE2D3*	0.003207972	0.539267016	23
3	*RAD21*	0.041471911	0.700680272	99	29	*H3-4*	0.006748015	0.591954023	58	55	*WAPL*	0.003045988	0.569060773	35	81	*NRG4*	0.002803111	0.542105263	23
4	*ERBB2*	0.033729856	0.647798742	97	30	*RBBP8*	0.013927344	0.609467456	57	56	*MATK*	0.005306963	0.56284153	35	82	*SPO11*	0.000919	0.533678756	22
5	*SMC3*	0.024540235	0.677631579	95	31	*FANCD2*	0.008613348	0.620481928	57	57	*PDS5A*	0.00140225	0.556756757	34	83	*CHTF18*	0.001374747	0.54787234	22
6	*H2AX*	0.013405078	0.673202614	91	32	*RAD50*	0.010785629	0.613095238	54	58	*UIMC1*	0.006850196	0.578651685	33	84	*CPNE3*	0.002769361	0.553763441	21
7	*EGFR*	0.037331182	0.686666667	90	33	*NBN*	0.005183089	0.605882353	54	59	*EREG*	0.004117006	0.565934066	32	85	*GDF15*	0.005372616	0.533678756	20
8	*SMC1A*	0.020461593	0.673202614	88	34	*ABL1*	0.013557634	0.598837209	52	60	*ESPL1*	0.00332041	0.569060773	31	86	*UBE2L3*	0.002088879	0.539267016	20
9	*PCNA*	0.010652542	0.647798742	86	35	*HSP90AA1*	0.01655376	0.620481928	52	61	*NIPBL*	0.001890953	0.556756757	30	87	*ELP1*	0.003535649	0.544973545	20
10	*ATM*	0.013749502	0.647798742	79	36	*ERBB3*	0.006454099	0.575418994	51	62	*PTPRU*	0.009620377	0.565934066	30	88	*CBLC*	0.001755647	0.530927835	20
11	*RPA2*	0.013683994	0.639751553	79	37	*ACD*	0.006502311	0.591954023	51	63	*EWSR1*	0.003118252	0.565934066	30	89	*GRAP2*	0.000645	0.52284264	19
12	*TP53*	0.01960873	0.651898734	78	38	*WRN*	0.004962086	0.588571429	49	64	*CDCA5*	0.001799483	0.56284153	30	90	*OSMR*	0.002061606	0.536458333	18
13	*RPA3*	0.013635104	0.651898734	77	39	*MSH6*	0.003215047	0.588571429	47	65	*GRB7*	0.00665337	0.556756757	28	91	*PTPN3*	0.001590995	0.5	18
14	*LMNB1*	0.014830546	0.63190184	77	40	*XRCC6*	0.008613721	0.602339181	46	66	*EZR*	0.006270099	0.56284153	28	92	*DOCK7*	0.001669995	0.530927835	18
15	*RPA1*	0.006205161	0.620481928	75	41	*GRB2*	0.008166818	0.581920904	46	67	*STUB1*	0.004058128	0.569060773	28	93	*HSP90B1*	0.001142712	0.539267016	17
16	*BLM*	0.011741794	0.635802469	72	42	*SRC*	0.002678368	0.544973545	46	68	*ERBIN*	0.00449116	0.553763441	28	94	*CHAMP1*	0.000441	0.50990099	16
17	*TERF2*	0.008332806	0.62804878	71	43	*CHEK2*	0.00613275	0.581920904	44	69	*HBEGF*	0.001807112	0.542105263	27	95	*RND1*	0.0010735	0.515	14
18	*STAG1*	0.015333907	0.62804878	69	44	*TINF2*	0.003513555	0.585227273	44	70	*ITGB4*	0.002182628	0.530927835	26	96	*UBXN1*	0.000841	0.52020202	14
19	*MRE11*	0.005677352	0.602339181	68	45	*LMNA*	0.008364602	0.609467456	43	71	*SEMA4D*	0.006309291	0.559782609	26	97	*SNTA1*	0.001653224	0.525510204	13
20	*PRKDC*	0.007213146	0.62804878	68	46	*REC8*	0.00768538	0.572222222	43	72	*PTPRK*	0.005509624	0.556756757	26	98	*DLG3*	0.000871	0.52020202	12
21	*TERF2IP*	0.016790304	0.616766467	66	47	*SMC1B*	0.002497762	0.550802139	42	73	*ELF3*	0.007946837	0.559782609	26	99	*ABCB5*	0.000539	0.52020202	10
22	*STAG2*	0.006697973	0.602339181	65	48	*BTC*	0.008564645	0.581920904	41	74	*CSTF1*	0.000825	0.530927835	25	##	*TTYH2*	0.00110062	0.495192308	9
23	*CDK4*	0.007709768	0.613095238	63	49	*PTK2B*	0.006537286	0.565934066	40	75	*RNF41*	0.006498725	0.556756757	24	##	*MRNIP*	0.001159343	0.5	9
24	*TOPBP1*	0.008330477	0.620481928	63	50	*ATRX*	0.004393549	0.578651685	39	76	*SIRT7*	0.004547676	0.559782609	24	##	*MUC16*	0.00014	0.495192308	6
25	*TERF1*	0.004011531	0.605882353	63	51	*PDS5B*	0.001953152	0.559782609	38	77	*SMC6*	0.002703696	0.542105263	23	##	*AC116366.2*	0.0000182	0.436440678	5
26	*POT1*	0.006785898	0.605882353	60	52	*RANBP2*	0.004291942	0.578651685	38	78	*PTK6*	0.002566824	0.533678756	23	##	*RAD21L1*	0	0.436440678	3

### Functional and pathway enrichment analyses

The functional enrichment pathways of the *RAD21*, *RAD50, BARD1* and *ERBB2* were investigated using DAVID software to identify significant GO categories and KEGG pathways. In the molecular function of GO; majority of genes involved in protein binding along with *ERBB2*, *RAD21* and *BARD1*. *ERBB2* and *RAD50* appears in both functions of identical protein binding and ATP binding. Likewise, protein heterodimerization activity showed *BARD1* and *ERBB2*. In the cellular component of GO; most genes are enriched in nucleus along with our four target genes. Cytosol shows *ERBB2* and *RAD21*. *RAD21*, *RAD50* and *BARD1* were also found in more functions and pathways involves in; cytoplasm, nucleoplasm, plasma membrane, site of double-strand break and macromolecular complex. In the biological process of GO results were; positive regulation of kinase activity involved both *ERBB2* and *RAD50*. Negative regulation of apoptotic process involved *ERBB2* and *BARD1*. DNA repair, cellular response to DNA damage stimulus, double-strand break repair, cell division and more are suggested to be regulates indirectly with our targeted genes. In the KEGG pathway enrichment analysis data demonstrated that; homologous recombination, *ERBB* signaling pathway, cell cycle, PI3K-Akt signaling pathway, microRNAs in cancer and pathways in cancer were associated with *RAD21*, *RAD50* and *BARD1* (Fig. 4B and Additional file 2: Table S3).

## Discussion

Several studies have highlighted the important role of homologous recombination factors (*RAD21*, *RAD50* and *BARD1*) in cancer progression, aggressiveness and genomic instability in many cancer types^22,24,28,43,65,66^. Though, these factors remain largely unexplored especially in bladder cancer. We recently highlighted the interplay between *ATM* (one of the homologous recombination factors) and *ERBB2* in bladder cancer patients^64^. In the current study, we observed a significant overexpression of *ERBB2* in bladder cancer tissues compared to normal. This overexpression was in agreement with many published studies that showed overexpression of *ERBB2* in solid cancers, serving as a prognostic and predictive biomarker especially in breast, gastric, colorectal and bladder cancer^67–70^. Kiss et al., reported that *ERBB2* amplification is not always associated with HER2 overexpression in bladder cancer, and HER2 overexpression was observed without gene amplification. Suggesting that both HER2 protein and *ERBB2* gene expressions are regulated by different mechanisms^71^. In this current investigation we examined *RAD21*, *RAD50* or/and *BARD1* co-expression with different status of *ERBB2* expression and assessed their prognostic and clinical significance in bladder cancer.

Published data reported that *RAD21* mRNA amplification correlates with gene copy number in grade 3 luminal, basal and HER2 subtypes of breast cancer. Also, RAD21 protein overexpression correlates strongly with gene amplification^27^. This overexpression was implicated in many cancer types and associate with poor outcomes in patients^22–26^. Similarly, *RAD21* mRNA was upregulated in bladder cancer tissues compared to normal tissues, also an increase in mRNA level was detected in late-stage bladder cancer cell lines^28^. In agreement with previous data, our data showed significant upregulation of *RAD21* expression in bladder cancer tissues compared to normal. To investigate the relation between *RAD21* and *ERBB2* expression, we sub-grouped all patients cohorts according to *ERBB2* status. Interestingly we found that *RAD21* mRNA level increase significantly in patients with low-*ERBB2* compared to patients with high-*ERBB2*. Furthermore, as Yu et al., indicated in their whole cohort *RAD21* expression alone did not influence survival significantly on the OS^28^. In this study, we confirmed this in OS and DFS, also in both subgroups of *ERBB2* cohorts. Interestingly we found high *RAD21* mRNA was linked to poor survival in the *ERBB2*-low cohort in the main MIBC cohort and confirmed it in both high grade and MIBC validation cohorts. Furthermore, we also found this trend in both the low tumor grade subgroup and the early tumor stage (Ta) subgroup of the NMIBC validation cohort. In contrast, *RAD21* low mRNA showed significant association with low tumor grade in *ERBB2*-high cohort. These findings suggest that additional data maybe required in the future to corroborate statistically the impact that *RAD21* plays in specific types of bladder cancer.

RAD50 is one of the key players in homologous recombination repair and telomere maintenance^29^. Literature is reporting that RAD50 high expression associated with aggressive high grade cystadenocarcinomas and low RAD50 linked to better progression free survival^72^. The aggressive phenotype and poor survival associated with high RAD50 expression at protein and transcriptomic levels was also reported in bladder, gastric, colorectal, rectal and ovarian cancers^73–76^. Hence the RAD50 factor role is yet to be elucidated in different cancer types, in the current study we first assessed the total *RAD50* mRNA expression level which was not altered in bladder cancer compared to normal tissues. Interestingly, following the subgrouping of cohort according to *ERBB2* status, we found that *RAD50* mRNA level increased significantly in patients with low-*ERBB2* compared to patients with high-*ERBB2*. This increase was translated to poor DFS for patients with high *RAD50* in the *ERBB2*-low cohort. This finding was confirmed in the MIBC and the high grade cohorts. Moreover, the same trend was seen in the low grade subgroup and the early tumor stage (Ta) subgroup of the NMIBC cohort. These findings further support our conclusion that *RAD50* mRNA level may have a poor prognostic role in *ERBB2*-Low bladder cancer patients, regardless of the grade or stage distribution of the cohort. Further significant associations between clinicopathological variables and *RAD50* at different *ERBB2* levels were not seen.

BARD1 is another player in the homologous recombination pathway, it was suggested that this role in DNA repair pathway is through direct interact between BARD1 and BRCA1^40,41^. Variants of *BARD1* gene were associated with many solid tumors^44–46,77^. Hawsawi et al., recently illustrated that high *BARD1* mRNA expression was associated with poor OS, relapse free survival and distant metastasis free survival in breast, ovarian and gastric cancer but not lung cancer^43^. In the current study, *BARD1* mRNA did not show any alteration in expression level between bladder cancer tissues and normal. Though, significant upregulation was observed in *BARD1* mRNA in patients with low-*ERBB2* compared to patients with high-*ERBB2* in all study cohorts. Interestingly this high *BARD1* mRNA was translated to poorer DFS in the whole cohort and in the *ERBB2*-low cohort in compared to patients with low *BARD1*, though no significant was detected when *ERBB2* expression was high. Based on our analysis, we have observed an association between high levels of *BARD1* mRNA expression and poor survival in the main cohort, as well as the validation MIBC cohort. Additionally, we found a similar trend in the NMIBC subgroups, particularly in the low grade and early tumor stage (Ta–T1) patients. This implies that *BARD1* mRNA may be a promising prognostic marker for bladder cancer patients, irrespective of the tumor grade or stage. In contrast, *BARD1* low expression was significantly associated with low tumor grade and non-papillary tumor shape in *ERBB2*-high patients. We also showed that *BARD1* mRNAs expression was independent prognostic factor for worse DFS in the *ERBB2*-low cohort, but not in *ERBB2*-high cohort. Our observations suggest the potential value of the expression pattern of *BARD1* at specific subtypes of bladder cancer.

As we highlighted the role of each homologous recombination factors (*RAD21*, *RAD50* or *BARD1*) to patients’ survival and cancer aggressiveness in bladder cancer, other groups studied these factors in different cancer types^22,24,28,43,65,66^. Here we also performed a co-expression analysis of these factors. Our data demonstrated that patients with low *RAD21*/low *RAD50* tumors along with low *ERBB2* expression had better survival outcome compared to those patients with high *RAD21*/high *RAD50* tumors. Also, high *RAD21*/high *BARD1* tumors had the worst survival in the whole cohort and *ERBB2*-low patients, but not in *ERBB2*-high patients. The high expression of either *RAD21*/*RAD50* or *RAD21*/*BARD1* in *ERBB2*-low cohort had a significant association with an increased chance of metastasis compared to the other combinations. Similarly, low *RAD50/*low *BARD1* mRNA expression showed better outcome in compared to high *RAD50/*high *BARD1* tumors in the whole cohort and *ERBB2*-low patients. High expression of *RAD50/BARD1* associated significantly with papillary tumor shape in *ERBB2*-low patients. Multivariate analyses data showed that *RAD50*/*BARD1* mRNA expression was independent prognostic factor for poor DFS in the *ERBB2*-low patients. Therefore, these homologous recombination potential biomarkers may play roles in predicting metastasis and survival in bladder cancer patients.

We next sought to investigate the interaction network between *RAD21*, *RAD50*, *BARD1* and *ERBB2* to provide deeper insight into the molecular mechanisms of these relations through identifying the most related genes network between our targets. Overlapping genes were identified with high physical interactions, co-expression, co-localization, genetic interactions, shared pathway and shared protein domains with *RAD21*, *RAD50*, *BARD1* and *ERBB2*. These genes include: *BRCA1, SMC3, H2AX, EGFR, SMC1A, PCNA, ATM, RPA2, TP53, RPA3, LMNB1, RPA1, BLM, TERF2, STAG1, MRE11, PRKDC, TERF2IP, STAG2, CDK4, TOPBP1, TERF1, POT1* and more. Centrality measure of this network indicates the importance of these intermediate genes to the interaction between our targets. This was followed with the functional and pathway enrichment analysis which showed majority of the overlapping genes with *ERBB2*, *RAD21* and *BARD1* involves in protein binding. *ERBB2* and *RAD50* factors appear in identical protein binding and ATP binding. Moncalian et al., showed how the motif signature is essential to ATP binding and biological function of RAD50^78^. Tarsounas et al., discussed how BARD1 and BRCA1 heterodimers through its E3 ubiquitin ligase activity, then the ability of this heterodimer to interact with other DNA damage response factors through the homologous repair pathway^40^. Our data suggested the involvement of both *BARD1* and *ERBB2* along with other overlapping genes in protein heterodimerization activity. We also found that many genes are enriched along with our four target genes in the nucleus, which agrees with other studies emphasizing our target genes functional role in localizing to the nucleus to participate in the DNA repair^25,79,80^. In addition, data illustrated that *ERBB2* and *RAD50* appear in the positive regulation of kinase activity. Similarly, the enriched results also identified *ERBB2* and *BARD1* are requires in the negative regulation of apoptotic process^43,81^. Altogether, a strong overlap of *ERBB2*-driven pathways was found with our homologous recombination factors, which may help define a signature to select bladder cancer patients who may benefit from targeted therapy and may use to evaluate drug response for patients.

## Conclusions

To our knowledge, this is the first time where the relationship between *RAD21*, *RAD50*, *BARD1* and *ERBB2* was highlighted in bladder cancer. This study provided novel findings and potential prognostic markers in this type of cancer. Importantly, here we showed that high *RAD21*, *RAD50* or *BARD1* mRNA expression in bladder cancer patients with low-*ERBB2* exhibit poor survival. In addition, gene expression of *BARD1* alone or in combination with *RAD50* acted as an independent prognostic factor for worst survival. We also identified several promising candidate genes between our targets which could be incorporated in tumor prognosis. The fact that this is a retrospective observational study is the main limitation of our work, therefore further analysis is needed. Additionally, we recognize that the median value method we used to divide the dataset into two groups based on expression levels may also have limitations due to the small sample size and limited clinical data available. In future studies, we plan to utilize more advanced methods that can accommodate larger sample sizes and more comprehensive clinical data. This is to better assess the clinical relevance of differentially expressed genes and identify potential biomarkers for bladder cancer prognosis. Also, the exact molecular mechanism between our homologous recombination targets and *ERBB2* still need to be investigated to improve prognosis and treatment efficacy in bladder cancer. Using bioinformatical analysis tools to find potential overlapping gene is a good step, though validating these finding with experimental test is a must to understand the mechanism.

## Supplementary Information

Supplementary Figures.

Supplementary Tables.

## Data Availability

All data analyzed during this study are from publicly available databases as indicated in the Materials and methods/Study cohorts and data analysis. TCGA data were downloaded from UALCAN portal (http://ualcan.path.uab.edu/index.html); [BLCA] and cBioPortal (https://www.cbioportal.org/); Bladder Cancer [TCGA, Cell 2017] and Bladder Cancer [MSK, J Clin Onco 2013]. From GEO database; Platform GPL570 [accession no. ‘GSE31684’] and Platform GPL6947 [accession no. ‘GSE48075’]. From ArrayExpress database (https://www.ebi.ac.uk/biostudies/arrayexpress) accession no. E-MTAB-4321 was used.

## Abbreviations

NMIBC: Non-muscle invasive bladder cancer
MIBC: Muscle-invasive bladder cancer
HER2/ERBB2: Human epidermal growth factor receptor 2
BRCA1: Breast cancer 1 gene
TCGA: The Cancer Genome Atlas
MSK: Memorial Sloan Kettering
GEO: The Gene Expression Omnibus
GO: Gene Ontology
KEGG: Kyoto Encyclopedia of Gene and Genomes
DAVID: Database for Annotation, Visualization and Integrated Discovery tool
OS: Overall survival
DFS: Disease-free survival
