# Supplementary material for: Homologous recombination mRNAs (RAD21, RAD50 and BARD1) have a potentially poor prognostic role in ERBB2-low bladder cancer patients
Source: Sci Rep. 2023 Jul 20;13:11738. doi: 10.1038/s41598-023-38923-y (PMC10359419; doi:10.1038/s41598-023-38923-y)
Supplement: Supplementary file 1 — Supplementary Figures. [file 41598_2023_38923_MOESM1_ESM.pptx]

## Slide 1
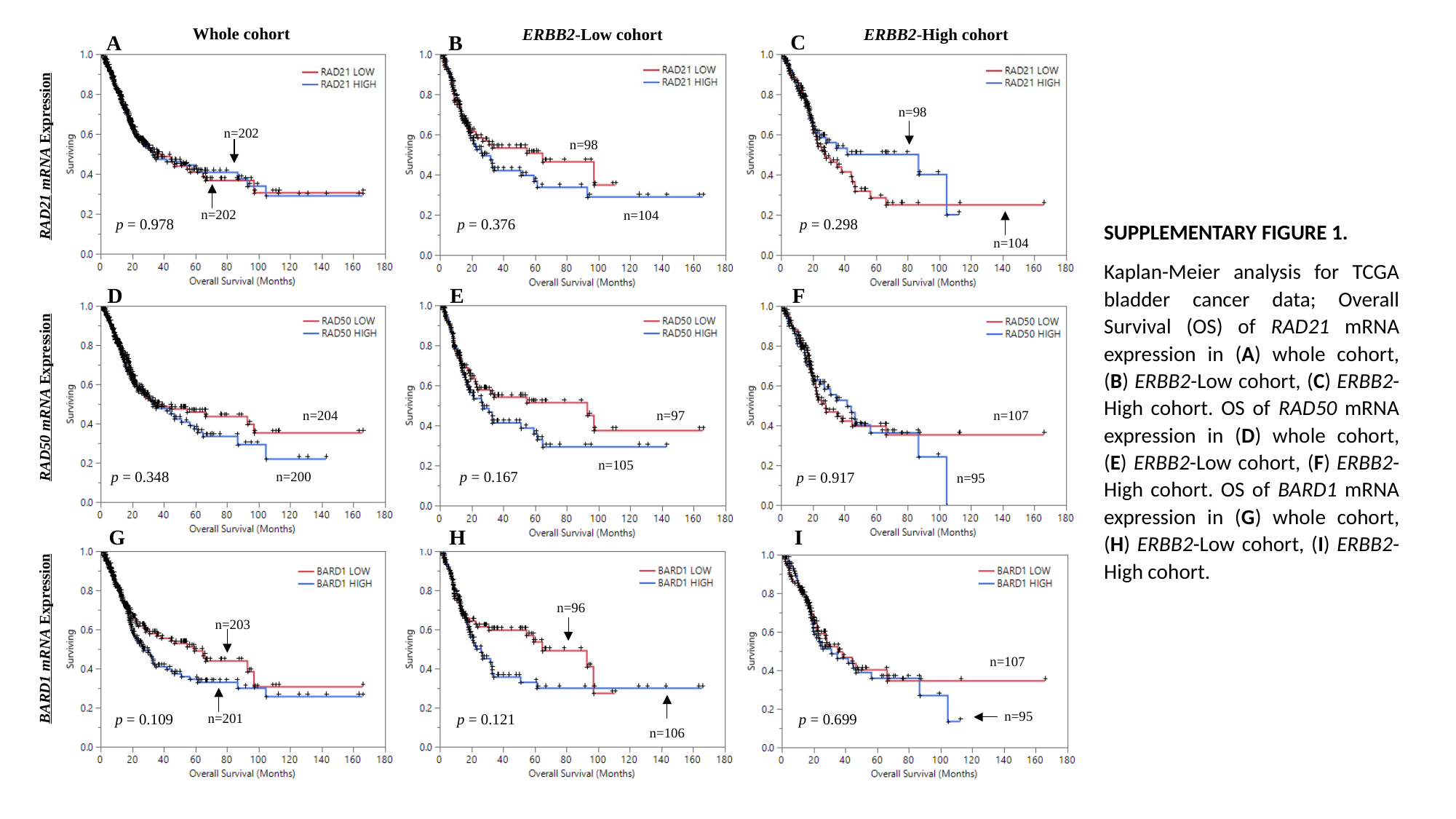

Whole cohort
ERBB2-High cohort
ERBB2-Low cohort
C
A
B
n=98
n=202
n=98
RAD21 mRNA Expression
n=202
n=104
p = 0.376
p = 0.298
p = 0.978
SUPPLEMENTARY FIGURE 1.
Kaplan-Meier analysis for TCGA bladder cancer data; Overall Survival (OS) of RAD21 mRNA expression in (A) whole cohort, (B) ERBB2-Low cohort, (C) ERBB2-High cohort. OS of RAD50 mRNA expression in (D) whole cohort, (E) ERBB2-Low cohort, (F) ERBB2-High cohort. OS of BARD1 mRNA expression in (G) whole cohort, (H) ERBB2-Low cohort, (I) ERBB2-High cohort.
n=104
F
D
E
RAD50 mRNA Expression
n=204
n=97
n=107
n=105
p = 0.167
p = 0.348
n=200
p = 0.917
n=95
G
H
I
n=96
n=203
BARD1 mRNA Expression
n=107
n=95
p = 0.121
p = 0.109
p = 0.699
n=201
n=106

## Slide 2
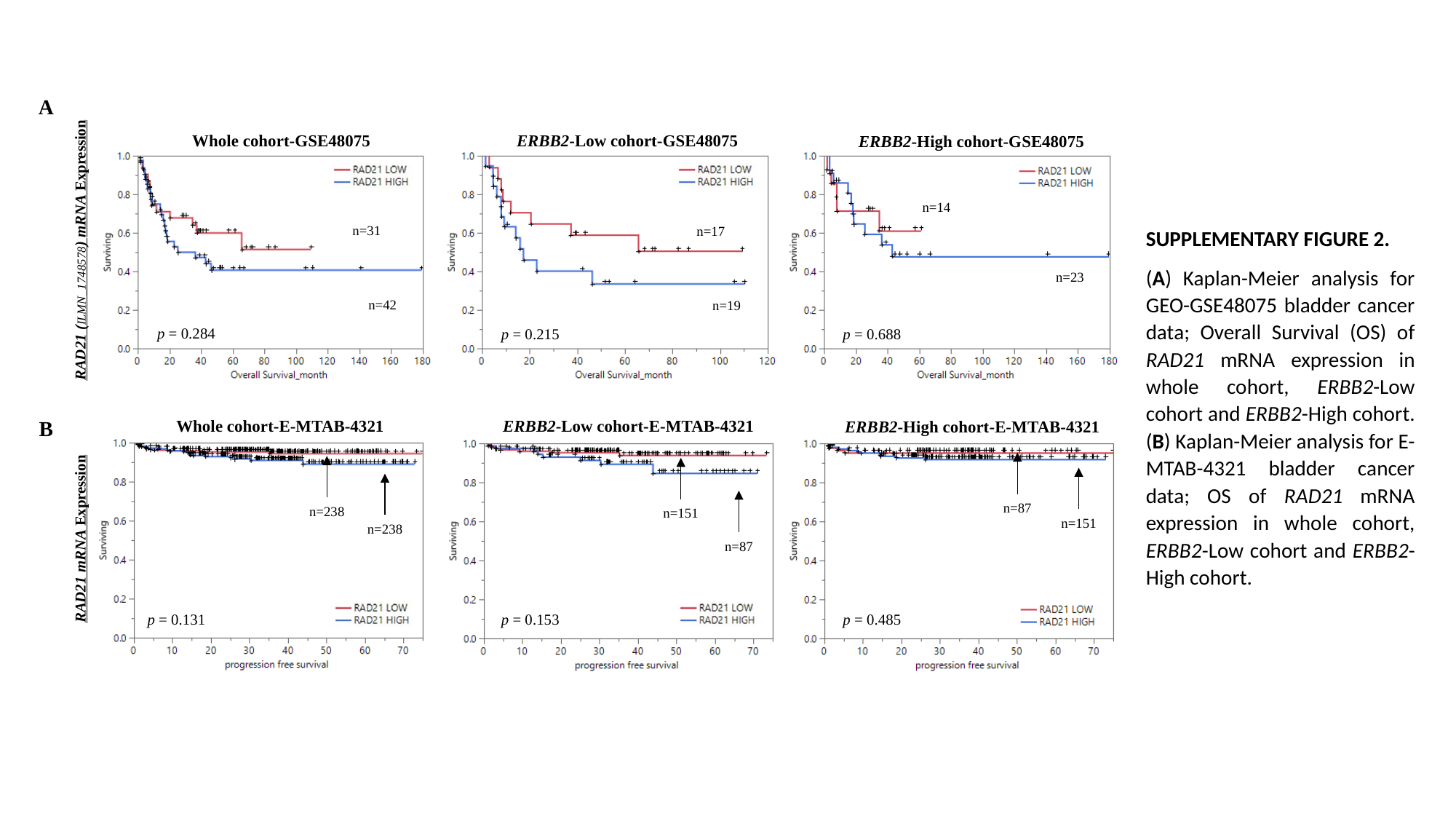

A
Whole cohort-GSE48075
ERBB2-Low cohort-GSE48075
ERBB2-High cohort-GSE48075
n=14
n=31
n=17
RAD21 (ILMN_1748578) mRNA Expression
n=23
n=42
n=19
p = 0.284
p = 0.688
p = 0.215
SUPPLEMENTARY FIGURE 2.
(A) Kaplan-Meier analysis for GEO-GSE48075 bladder cancer data; Overall Survival (OS) of RAD21 mRNA expression in whole cohort, ERBB2-Low cohort and ERBB2-High cohort. (B) Kaplan-Meier analysis for E-MTAB-4321 bladder cancer data; OS of RAD21 mRNA expression in whole cohort, ERBB2-Low cohort and ERBB2-High cohort.
B
Whole cohort-E-MTAB-4321
ERBB2-Low cohort-E-MTAB-4321
ERBB2-High cohort-E-MTAB-4321
n=87
n=238
n=151
n=151
n=238
RAD21 mRNA Expression
n=87
p = 0.485
p = 0.131
p = 0.153

## Slide 3
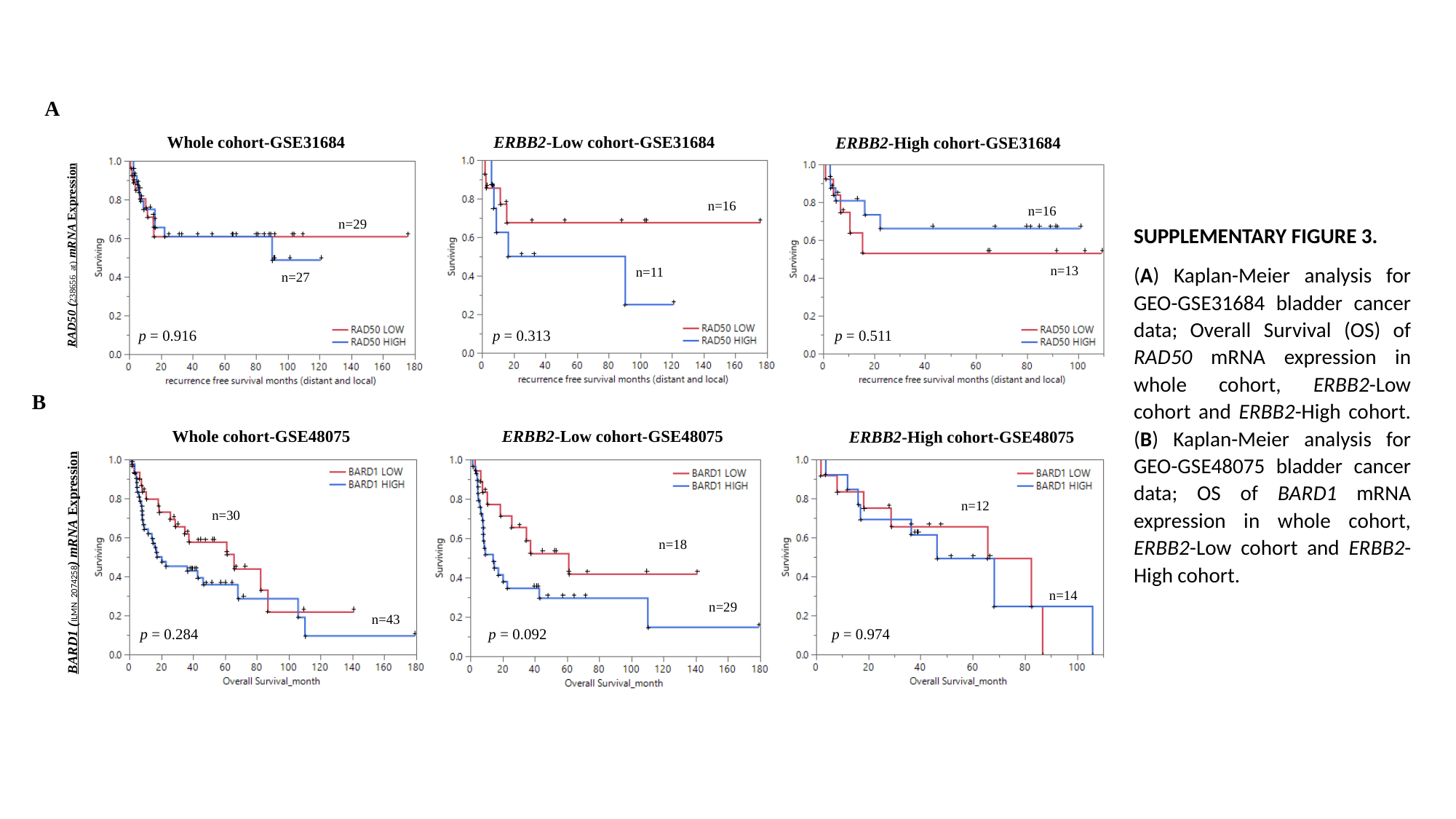

A
Whole cohort-GSE31684
ERBB2-Low cohort-GSE31684
ERBB2-High cohort-GSE31684
n=16
n=16
n=29
RAD50 (238656_at) mRNA Expression
n=13
n=11
n=27
p = 0.511
p = 0.916
p = 0.313
SUPPLEMENTARY FIGURE 3.
(A) Kaplan-Meier analysis for GEO-GSE31684 bladder cancer data; Overall Survival (OS) of RAD50 mRNA expression in whole cohort, ERBB2-Low cohort and ERBB2-High cohort. (B) Kaplan-Meier analysis for GEO-GSE48075 bladder cancer data; OS of BARD1 mRNA expression in whole cohort, ERBB2-Low cohort and ERBB2-High cohort.
B
Whole cohort-GSE48075
ERBB2-Low cohort-GSE48075
ERBB2-High cohort-GSE48075
n=12
n=30
n=18
BARD1 (ILMN_2074258) mRNA Expression
n=14
n=29
n=43
p = 0.284
p = 0.092
p = 0.974

## Slide 4
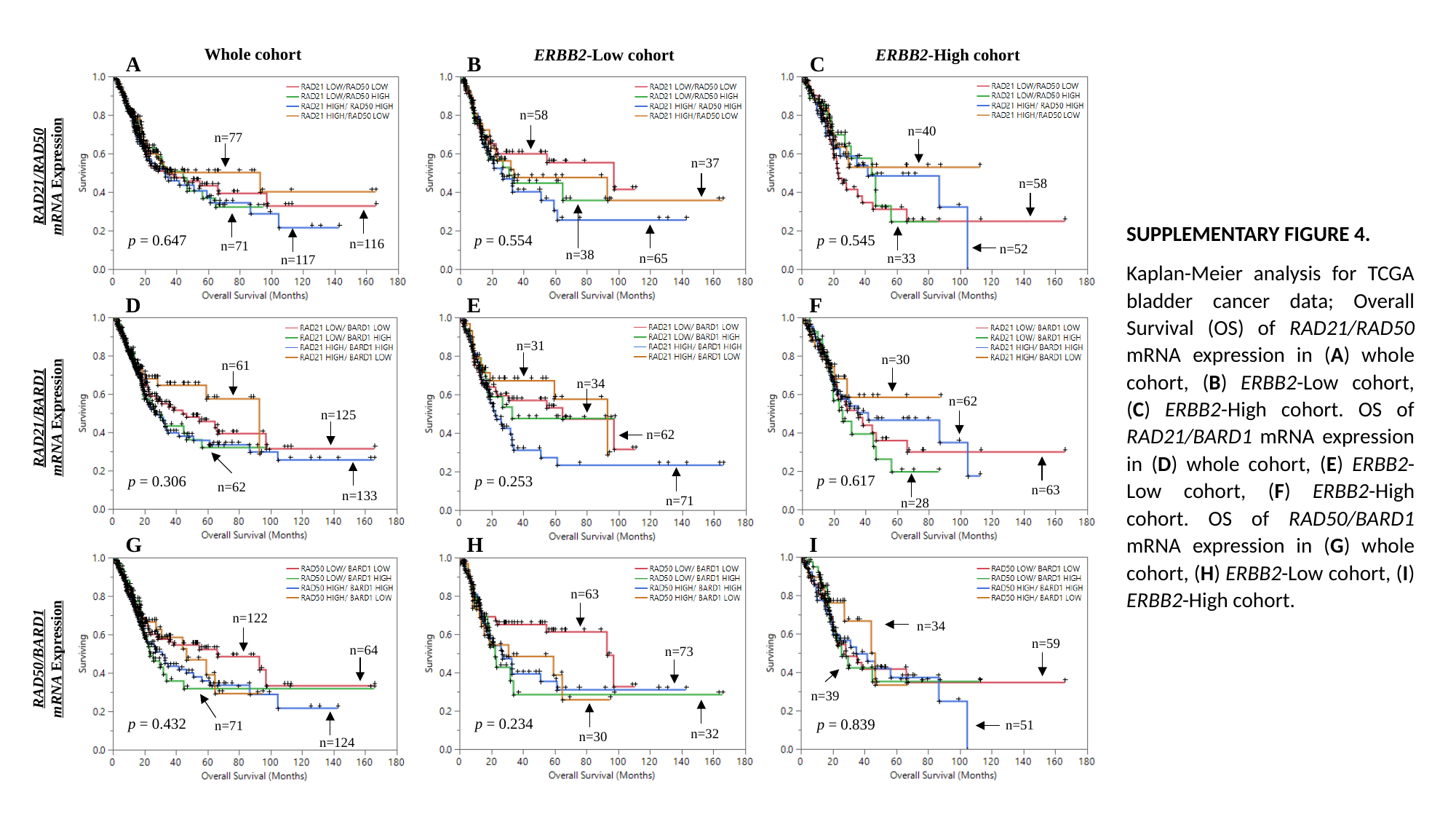

Whole cohort
ERBB2-High cohort
ERBB2-Low cohort
A
B
C
n=58
n=40
n=77
n=37
RAD21/RAD50
mRNA Expression
n=58
SUPPLEMENTARY FIGURE 4.
Kaplan-Meier analysis for TCGA bladder cancer data; Overall Survival (OS) of RAD21/RAD50 mRNA expression in (A) whole cohort, (B) ERBB2-Low cohort, (C) ERBB2-High cohort. OS of RAD21/BARD1 mRNA expression in (D) whole cohort, (E) ERBB2-Low cohort, (F) ERBB2-High cohort. OS of RAD50/BARD1 mRNA expression in (G) whole cohort, (H) ERBB2-Low cohort, (I) ERBB2-High cohort.
p = 0.554
p = 0.545
p = 0.647
n=116
n=71
n=52
n=38
n=65
n=33
n=117
D
E
F
n=31
n=30
n=61
n=34
n=62
RAD21/BARD1
mRNA Expression
n=125
n=62
p = 0.617
p = 0.306
p = 0.253
n=62
n=63
n=133
n=71
n=28
G
H
I
n=63
n=122
n=34
n=59
RAD50/BARD1
mRNA Expression
n=64
n=73
n=39
p = 0.234
p = 0.432
p = 0.839
n=51
n=71
n=32
n=30
n=124
